# Unveiling the Impacts of Cashew Nuts on Oxidative Stress in Rats: A Systematic Review

**DOI:** 10.3390/antiox14040441

**Published:** 2025-04-07

**Authors:** Roque Ribeiro da Silva Júnior, Vinicius Ilei Oliveira Rodrigues, Camila Fernandes Maia de Carvalho, Márcio Matheus Barros Moura, Deymisson Damitene Martins Feitosa, Emanuel Kennedy Feitosa Lima, Ariel Moraes de Andrade, Joel Freires de Alencar Arrais, Larissa Nayara de Souza, Maria Irany Knackfuss, José Rodolfo Lopes de Paiva Cavalcanti, Thales Allyrio Araújo De Medeiros Fernandes, Marcos Antônio Pereira dos Santos, Ivana Alice Teixeira Fonseca, Adalberto Veronese da Costa, Glêbia Alexa Cardoso

**Affiliations:** 1 Postgraduate Program in Health and Society, State University of Rio Grande do Norte, Mossoró 59607-360, Brazil; vinicius.rodrigues@uninassau.edu.br (V.I.O.R.); camilla_fndes@hotmail.com (C.F.M.d.C.); arielandrade702@alu.uern.br (A.M.d.A.); joel20241002093@alu.uern.br (J.F.d.A.A.); larissa20241002128@alu.uern.br (L.N.d.S.); mariaknackfuss@uern.br (M.I.K.); rodolfolopes@uern.br (J.R.L.d.P.C.); thalesallyrio@uern.br (T.A.A.D.M.F.); ivanateixeira@uern.br (I.A.T.F.); adalbertoveronese@uern.br (A.V.d.C.); 2 Multicenter Postgraduate Program in Physiological Sciences, State University of Rio Grande do Norte, Mossoró 59607-360, Brazil; 3 Department of Veterinary Medicine, Federal Rural University of Semi-Arid, Mossoró 59625-900, Brazil; marcio.moura@alunos.ufersa.edu.br; 4 Multicenter Postgraduate Program in Biochemistry and Molecular Biology (PMBqBM), Rio Grande do Norte State University, Mossoró 59607-360, Brazil; deymisson@usp.br (D.D.M.F.); emanuel.lima@ufersa.edu.br (E.K.F.L.); 5 Department of Physical Education, Federal University of Piauí, Teresina 64049-550, Brazil; marcosedfisio@gmail.com

**Keywords:** *Anacardium*, rats, oxidative stress, antioxidant, reactive oxygen species, redox balance

## Abstract

Introduction: The fruit of the cashew tree, known as cashew, is accompanied by the fleshy extension of its stem, referred to as the cashew nut. Rich in phenolic compounds, such as phenolic acids, anthocyanins, flavonoids, carotenoids, polyphenols, as well as vitamins C and E, the cashew nut exhibits antioxidant properties. Objective: This systematic review investigated the effects of cashew nuts on oxidative stress in rats. Methodology: The study followed PRISMA guidelines and was registered in PROSPERO. Searches were conducted in the Medline (PubMed), EMBASE, BVS, MedRxiv, Science Direct, Scopus, and Web of Science databases. Experimental studies with rats as the target population, evaluating the effects of cashew nut supplementation on oxidative stress, antioxidant enzymatic activities, and inflammatory markers, were included. Exclusion criteria comprised dissertations, reviews, expert opinions, duplicates, and preprints. Results: Five studies published between 2018 and 2022 were included, all utilizing cashew nut supplementation as the intervention. The results demonstrated a significant reduction in oxidative stress, an increase in the activity of antioxidant enzymes, such as SOD and catalase, and a decrease in inflammatory markers, including TNF-α and IL-1β. The most effective dose was 100 mg/kg/day, yielding consistent results across studies. Conclusion: Cashew nuts show potential for reducing oxidative stress, mitigating inflammation, and enhancing antioxidant defenses in rats. However, further clinical studies are required to better explore their benefits in humans, a field that remains less studied compared to other types of nuts.

## 1. Introduction

The plant family known as *Anacardiaceae* comprises approximately 77 genera and about 700 species. These plants have a particular affinity for rocky, sandy, and clay soils and are frequently found in tropical and subtropical regions. Within this family, the genus *Anacardium* consists of 20 distinct species, with *Anacardium occidentale* L. (cashew tree) being the most recognized and widely cultivated [1,2].

The root system of a mature cashew tree is composed of a robust main root and an extensive, well-developed network of lateral and adventitious roots that significantly expand from the seed. These plants tend to produce their best yields when grown under favorable climatic conditions. In the semi-arid tropical regions of Africa, India, Sri Lanka, Brazil, and Southeast Asia, cashew nuts are commercially cultivated. In 2010, the global production of cashew nuts reached 3.6 million tons, harvested from 4.4 million hectares, with Brazil being one of the main commercial producers [1].

The fruit of the cashew tree, known as the cashew apple, is accompanied by the fleshy extension of the stem, called the cashew nut. This nut is rich in phenolic compounds, including phenolic acids, anthocyanins, flavonoids, carotenoids, polyphenols, as well as vitamins C and E, which possess anti-inflammatory and antioxidant properties [3].

Oxidative stress (OS) is a physiological condition characterized by an imbalance between oxidant compounds and the capacity of the body’s antioxidant defense systems [4]. This imbalance results in the formation of reactive oxygen species (ROS) and reactive nitrogen species (RNS), which are byproducts of oxygen and nitrogen metabolism [5]. Therefore, OS represents a disturbance in the production of ROS and RNS, requiring an acute and efficient antioxidant response [6].

Antioxidant defenses are physiological systems that counterbalance oxidative stress, forming, along with it, what is known as redox balance. Antioxidants act by inhibiting and reducing the damage caused by free radicals and/or non-radical reactive species. They can act through different mechanisms: preventing the formation of free radicals or non-radical species, blocking their deleterious actions, or promoting the repair and reconstitution of damaged biological structures [7].

This defense system is divided into enzymatic and non-enzymatic components. The non-enzymatic system comprises a variety of antioxidant substances, which can be of endogenous or dietary origin. Antioxidants are defined as any substance that, present in lower concentrations than the oxidizable substrate, is capable of delaying or inhibiting its oxidation effectively. These substances can act directly, neutralizing the action of free radicals and non-radical species, or indirectly, participating in enzymatic systems with antioxidant capacity [8].

The objective of this study was to investigate the effects of cashew nuts on oxidative stress in rats.

## 2. Methodology

### 2.1. Study Type

This study is characterized as a systematic review, following the methodology guided by the Preferred Reporting Items for Systematic Reviews (PRISMA) guidelines developed by Page et al. [9]. The entire protocol for conducting the review was registered with the National Institute for Health Research, in the International Prospective Register of Ongoing Systematic Reviews—PROSPERO, under the registration number CRD42023446200.

### 2.2. Databases

A search was conducted from November 2022 to July 2023 in the following databases: Medline (PubMed), EMBASE (Elsevier), Virtual Health Library (BVS), Library, MedRxiv, Science Direct, Scopus, and Web of Science. The search was limited to a period of time between 2018 and 2022.

### 2.3. Search Strategy

The search strategy was based on the PICOT question, where “P” refers to the population of the study, which involved animals, specifically rats of any lineage or family. The letter “I” represents the intervention, which is exposure to a cashew nut diet. The letter “C” indicates the comparison group, which includes nothing, placebo, or other interventions. The English term “Outcome,” represented by the letter “O,” refers to the reduction of oxidative stress, pro-inflammatory substances, and the enhancement of antioxidant defense. Finally, the letter “T” stands for the type of study, which includes experimental studies. The main search terms were retrieved from the Medical Subject Headings (MeSH) and included Anacardium, Oxidative Stress, and Rats, combined using the Boolean operator AND. The combinations used were: Anacardium and Oxidative Stress AND Rats, Rats AND Anacardium AND Oxidative Stress, and Oxidative Stress AND Anacardium AND Rats. This search strategy was applied with high sensitivity or systematically, as shown in Table 1. The same search strategy was used for all the databases.

### 2.4. Eligibility Criteria

The study included experimental studies with rodents as the study population, specifically rats, where the intervention involved a cashew nut diet and comparisons were made with other techniques, placebo, or nothing. The outcome of interest was to understand the benefits of the proposed intervention on the animals. Exclusion criteria encompassed the following: completion of course works, expert opinions, duplicates, reviews, editorials, and preprints.

### 2.5. Article Selection

After the high-sensitivity search in the databases, article selection was carried out through Rayyan QCRI, which facilitates blind assessments of systematic reviews. Three evaluators were registered in the system, and the captured articles from the high-sensitivity search were assigned to them for analysis. The technical critics of the articles were divided into two stages. In the first stage, two evaluators, R.R.S.J. and V.I.O.R., assessed the articles, and in case of divergence, the article was reviewed by the senior expert evaluator, G.A.C., in the second stage, who made the final decision on article selection. After applying the filters based on the eligibility criteria, the selection process was divided into two stages. The first stage involved the initial reading of the title and abstract of the articles in the software, and to proceed to the selection stages, an article needed at least one evaluator’s acceptance. The second stage involved the full reading of the article, and for an article to be included in the study, it required the acceptance of two evaluators.

### 2.6. Bias and Methodological Quality Assessment

The evaluative screening was conducted using the Systematic Review Center for Laboratory Animal Experimentation (RoB/SYRCLE), a methodological assessment tool for animal experimental studies developed by The Cochrane Collaboration. It contains ten items related to selection bias, performance bias, detection bias, attrition bias, reporting bias, and other biases. Half of these items align with the Cochrane tool. Studies deemed to have a high risk of bias were removed from the results.

### 2.7. Flowchart

**Figure 1:**
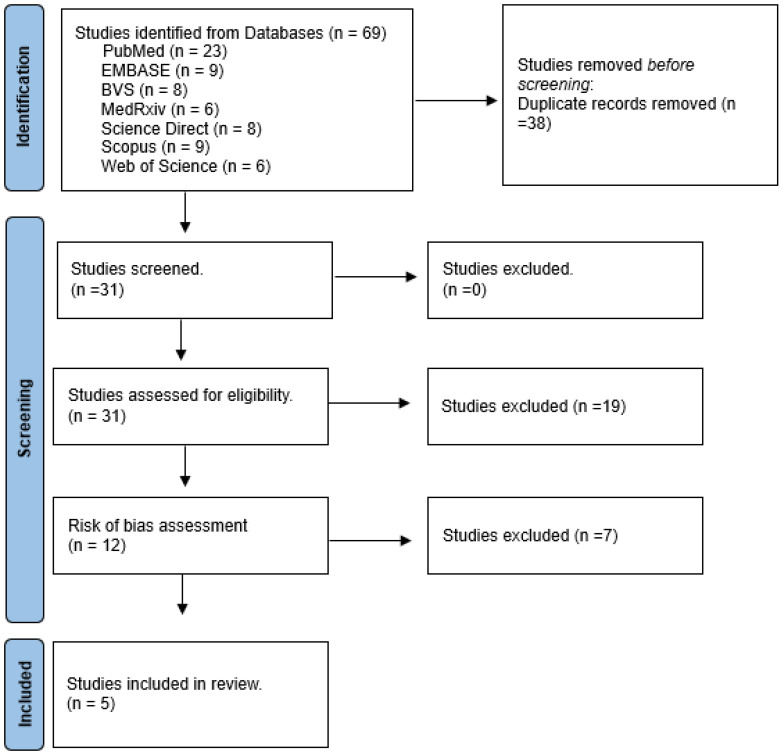
Flowchart.

A total of 69 articles were found on the proposed theme in the aforementioned databases. After removing duplicate articles using the Rayyan program, 38 journals were excluded. Consequently, 31 articles remained and underwent eligibility criteria assessment, resulting in the exclusion of 19 articles. After evaluating the risk of bias through Syrcle, 12 articles were left, and those with a high risk of bias were excluded, resulting in 5 articles that were included in the final results, as shown in Figure 1.

Among the observed conditions, it is evident that experimental studies focusing on cashew nuts (*Anacardium occidentale* L.) are limited in the databases mentioned in the study’s methodology, particularly regarding the relationship between cashew nuts and oxidative stress. Moreover, a significant portion of the studies was excluded for not meeting the eligibility criteria.

## 3. Results

All the articles included in the results section were experiments conducted on rats. Additionally, all studies involved supplementation with cashew nuts as an intervention. The temporal range of these studies spanned from 2018 to 2022, and they were all published in the English language, evaluating their impacts on oxidative stress and other physiological markers.

It is important to highlight the scarcity of literature on cashew nuts and their relationship with oxidative stress. A significant variability is observed in the methods and materials used, dosage, routes of administration, isolated substances, and other compounds from cashew nuts, studied tissues, experimental period, and study groups. This heterogeneity in the experimental conditions does not allow for providing clear and cohesive evidence on the efficacy of cashew nuts in controlling oxidative stress, but it suggests the need to explore the potential effects of cashew nuts and their isolated compounds.

In this context, we present an analysis of the following situations: The research conducted by Medeiros-Linard et al. [10] investigated the effects of anacardic acids (AAs), compounds found in cashew nuts, on controlling behavioral alterations and oxidative stress in rodent models treated with rotenone to simulate Parkinson’s disease. The focus was on the isolated substance anacardic acids, which are alkylphenols present in cashew nuts.

The experimental models involved rats divided into groups: untreated control group, group treated with rotenone only, group treated with AAs associated with rotenone, and group treated with AAs only. The groups treated with AAs and rotenone received oral doses of AAs at the following concentrations: 1, 3, 10, 25, 50, and 100 mg/kg/day, followed by subcutaneous administration of rotenone (3 mg/kg/day). The group treated with rotenone only received a solution of rotenone (3 mg/mL, via subcutaneous injection). The group treated exclusively with AAs received an oral dose of 100 mg/kg/day to evaluate the direct effects of these compounds.

The experiment lasted for 5 consecutive days, with administrations conducted in the morning between 8:00 and 9:00 a.m. One hour after AA administration, the animals in the combined treatment groups received rotenone. At the end of the experimental period, biological markers, behavioral changes, and lipid peroxidation levels in the substantia nigra of the brain were evaluated, given the relevance of this region to Parkinson’s disease.

The behavioral tests included assessments of the animals’ movement (locomotion), memory, and coordination. Oxidative stress was evaluated by measuring the activity of the antioxidant enzyme superoxide dismutase (SOD), followed by an analysis of gene expression for this same antioxidant enzyme at the cellular level, both in the basal ganglia and the cerebral cortex. It was observed that the isolated use of AAs reduced and prevented oxidative stress, acting primarily at the cellular level through cytoplasmic mechanisms. Regarding the experimental results, it is important to highlight that they focused on five main areas: memory performance, motor activity, lipoperoxidation, SOD activity, and gene expression. The animals treated with rotenone exhibited significant reductions in these activities. In contrast, the groups treated with AAs, particularly at dosages between 25 and 100 mg/kg/day, showed significant improvement in the changes caused by rotenone.

With respect to memory, rotenone caused severe impairment, leading to a 90% reduction in memory capacity. Consequently, doses of 10 mg/kg/day of AAs were administered, and the results indicated a restoration of memory capacity to normal levels. For motor activity, the Rotarod test was used for evaluation. The groups treated with rotenone displayed a 90% reduction in the time the rats were able to remain on the equipment. However, the animals treated with AAs showed significant improvement, with dosages ranging from 3 mg/kg/day to 100 mg/kg/day. The results indicated a progressive improvement with increasing dosages, with the highest dose (100 mg/kg/day) fully restoring motor coordination to normal levels.

In terms of oxidative stress markers, which were the main focus of the study, rotenone caused a drastic 26-fold increase in lipoperoxidation in brain regions, particularly in the substantia nigra, cortex, and striatum, compared to the untreated control group. The administration of different doses of AAs demonstrated effects in reducing oxidative stress. Nearly all dosages produced favorable results, with doses between 10 and 100 mg/kg/day showing protective effects, reducing oxidative stress to levels close to those of the control group. Regarding SOD activity, the groups that received AAs exhibited an increase in the activity of this enzyme in the brain structures studied, leading to an enhanced antioxidant defense in both the cortex and the striatum. The most effective dosages ranged between 25 and 100 mg/kg/day. As for gene expression, a significant increase was observed in the groups treated with AAs. The expression of the SOD-1 and SOD-2 genes in brain tissues increased by 2490 and 190 times, respectively, indicating a strong antioxidant response in the analyzed tissues.

Unlike the previous study, the subsequent research did not utilize isolated substances but involved the consumption of roasted cashew nuts, observing a convergence in lipid peroxidation levels. The experiments conducted by Akomolafe and Asowata-Ayodele [11] investigated the neuroprotective activity of roasted cashew nut supplementation in relation to brain alterations induced by cisplatin (CIS). Rats were used and divided into groups, with one hundred grams of roasted cashew nuts administered, and the appropriate percentages allocated to each study group. Additionally, the substance cisplatin was used. Thus, the groups were divided into a control group, which did not receive any intervention related to the proposed cashew nut treatment, and therapeutic groups subjected to the following treatment sequences: 7 mg/kg of CIS combined with 10% and 20% roasted cashew nuts, with the intervention lasting up to 28 days. The evaluation of neuroprotective markers was conducted through enzymes related to brain function, such as adenosine deaminase, along with oxidative stress and antioxidant defense analyses, including the measurement of reactive oxygen species (ROS), total antioxidant capacity (TAC), peroxidases, and other antioxidant enzymes involved in oxidative stress. These analyses were performed on the brain tissues of the rats.

In terms of the results, four main axes stand out: acetylcholinesterase (AChE), adenosine deaminase (ADA), oxidative stress, and histopathological analyses. Regarding AChE activity, a statistically significant increase (*p* < 0.05) was observed in the groups that received only CIS. In the groups treated with cashew nuts, there was a marked reduction in AChE activity, with the greatest reduction seen in the groups that received 20% cashew nuts. As for the ADA marker, cisplatin also promoted a significant increase in the activity of this enzyme. However, in the groups supplemented with 10% and 20% cashew nuts, there was a statistically significant reduction (*p* < 0.05), with the important note that increased ADA is associated with memory impairment. Regarding the oxidative stress marker, one of the main objectives of this review, the administration of CIS caused a significant increase in reactive oxygen species (ROS) levels and thiobarbituric acid reactive substances (TBARS), both indicators of oxidative damage. In the groups supplemented with 10% and 20% cashew nuts, a significant reduction in oxidative stress was observed. Moreover, other physiological markers related to antioxidant defense, such as SOD, catalase (CAT), glutathione-S-transferase (GST), and glutathione peroxidase (GPx), showed significant increases in the groups supplemented with 10% and 20% cashew nuts.

Finally, histopathological analyses of the tissues revealed that animals treated with CIS showed significant neuronal degeneration, as well as alterations in nuclei and the granular layer of the brain. In contrast, animals supplemented with 10% and 20% cashew nuts, especially those in the 20% group, exhibited normal brain structures when compared to the control group. Thus, the results indicate that a diet enriched with cashew nuts provides significant protection against the neurotoxic effects of cisplatin, reducing oxidative stress and preserving neuronal integrity.

In the experiments conducted by Fusco et al. [12], the impacts of oral cashew nut consumption were evaluated in rats with intestinal ischemia/reperfusion (I/R) injuries. Sprague–Dawley rats were divided into groups: a sham group (animals subjected to surgery without I/R induction and treated with a control), an I/R + control group (animals subjected to I/R and treated with a control), and groups subjected to I/R injury and cashew nut consumption at approximately 100 mg/kg. The research findings were organized into five main areas: reduction in mortality and blood pressure, antioxidant capacity, oxidative stress, neutrophil expression and adhesion, and signaling pathways. Regarding mortality and blood pressure, occlusion of the superior mesenteric artery resulted in 100% mortality in the animals treated with the vehicle after 4 hours of reperfusion. However, cashew nut administration (100 mg/kg) significantly reduced this mortality. Additionally, the drop in mean arterial pressure observed after reperfusion was significantly lower in the animals treated with cashew nuts.

In terms of neutrophil activity and adhesion markers, the expression of ICAM-1 and P-selectin was significantly elevated in the animals treated with the vehicle. However, in the groups supplemented with cashew nuts (100 mg/kg), there was a reduction in these expressions. Furthermore, the activity of myeloperoxidase (MPO), a marker of neutrophil infiltration, was also decreased with cashew nut supplementation.

Regarding oxidative stress, cashew nut administration resulted in a significant reduction in oxidative stress and protein carbonyl content (PCC), both indicators of oxidative damage. This effect was accompanied by a decrease in immunoreactivity for nitrotyrosine and the expression of PARP protein, markers of exacerbated oxidative damage. In terms of antioxidant capacity, the animals treated with cashew nuts showed a significant restoration of antioxidant enzyme activities, such as catalase (CAT), superoxide dismutase (SOD), glutathione-S-transferase (GST), and glutathione peroxidase (GPx), which had been reduced by I/R injury in the animals treated with the vehicle. The levels of these enzymes were restored to values similar to those of the control group.

Finally, it was observed that cashew nuts positively modulated the signaling pathways of Nrf2/HO-1 and reduced the activation of the NF-kB pathway, which is responsible for the inflammatory response. The expression of Nrf2 was increased, promoting greater antioxidant defense, while the translocation of NF-kB to the nucleus was reduced, leading to lower activation of inflammatory mediators. These results indicate that cashew nuts, at a dose of 100 mg/kg, have a potent protective effect against ischemia/reperfusion-induced injuries, with a reduction in oxidative stress and an increase in antioxidant activity, offering a promising approach for the prevention of ischemic diseases.

Similarly, the experiments conducted by D’Amico et al. [13] aimed to evaluate the anti-inflammatory and antioxidant effects of cashew nuts in a rat model of hyperhomocysteinemia (HHcy) induced by oral methionine administration. Sprague–Dawley rats were used and divided into four groups: sham + vehicle (rats that received saline solution and were treated with vehicle), sham + cashew nuts (rats that received saline solution and were treated with cashew nuts, 100 mg/kg), methionine + vehicle (rats that received methionine, 1 g/kg, and were treated with vehicle), and methionine + cashew nuts (rats that received methionine and were treated with cashew nuts, 100 mg/kg). The tissues studied were liver, colon, and kidneys, and the treatment was administered orally for 30 days.

The markers analyzed included homocysteine (Hcy) levels, total cholesterol, ALT, AST, ALP, LDH, creatinine, SOD, GSH, CAT, malondialdehyde (MDA), TNF-α, IL-1β, histological analysis of liver, kidney, and colon tissues, nitrotyrosine, PARP, NF-κB, NRF-2, HO-1, expression of Bax and Bcl-2, as well as the TUNEL assay to detect apoptotic fragments.

Regarding the results, a division was made between biochemical markers, oxidative stress markers, inflammatory cytokines, histological analyses, and nitrosative stress and apoptosis. In the biochemical analysis, rats with methionine-induced HHcy showed increased levels of total cholesterol, ALT, AST, ALP, LDH, and plasma creatinine. In contrast, the groups that received cashew nut supplementation showed a statistically significant reduction in these markers (*p* < 0.05).

When observing the main markers of the study, important results were found related to oxidative stress and antioxidant capacity. Animals supplemented with cashew nuts showed reduced levels of HHcy, as well as a significant reduction in lipid peroxidation levels, measured by MDA (*p* < 0.001). There was also a significant increase (*p* < 0.001) in the antioxidant enzymes SOD, CAT, and GSH. Additionally, the levels of pro-inflammatory cytokines TNF-α and IL-1β were significantly reduced (*p* < 0.001).

Finally, histological analyses showed that animals treated with cashew nuts presented a reduction in necrosis and cellular infiltration in liver and colon tissues. In this same context, the evaluation of nitrosative stress and apoptosis revealed that cashew nuts reduced positive staining for nitrotyrosine and PARP. Moreover, there was a significant reduction in apoptosis in the tissues treated with cashew nuts. In conclusion, cashew nuts demonstrated a strong protective effect against oxidative stress, inflammation, and tissue damage induced by hyperhomocysteinemia, making them a promising intervention for related inflammatory conditions.

Finally, in the experiment conducted by Cordaro et al. [14], the objective was to evaluate the anti-inflammatory and antioxidant effects of cashew nuts in an acute experimental model of carrageenan-induced paw edema in rats. One hundred milligrams per kilogram of cashew nuts were administered orally, with Sprague–Dawley rats divided into groups: carrageenan + vehicle (rats subjected to carrageenan-induced edema and treated with vehicle), carrageenan + cashew nuts (rats subjected to carrageenan-induced edema and treated with cashew nuts, 100 mg/kg, 30 min before induction), and sham group (rats undergoing the same surgical procedures without carrageenan injection). The studied tissues were the paw and blood for histological and biochemical analyses. The analyses included myeloperoxidase (MPO) activity, malondialdehyde (MDA) levels, nitrite/nitrate levels, evaluation of antioxidant enzymes SOD, CAT, and GSH, serum levels of TNF-α, IL-6, IL-1β, and IL-10, histological examination of paw tissues, thermal hyperalgesia tests (Plantar Test), mechanical allodynia (Von Frey Test), and expression of 5-LOX and Cox-2.

The results of the experiment were organized into key areas: edema and pain, MPO and MDA activity, nitric oxide synthesis, inflammatory cytokines, oxidative stress, and histological changes. Regarding edema and pain, it was observed that supplementation with cashew nuts, at a dose of 100 mg/kg, actively reduced both. Additionally, in terms of MPO and MDA activity, cashew nuts significantly reduced MPO activity, an indicator of neutrophil infiltration, and MDA levels, a marker of lipid peroxidation. These reductions suggest the effectiveness of cashew nuts in decreasing oxidative stress and inflammation. MPO and MDA levels, which were elevated after carrageenan (CAR) injection, were significantly reduced following cashew nut administration (*p* < 0.05 vs. CAR).

In the context of oxidative stress, it was also found that cashew nuts significantly reduced nitrite/nitrate levels in paw tissues, key indicators of nitric oxide (NO) synthesis during inflammatory events. The treatment effectively lowered these levels (*p* < 0.01 vs. CAR), contributing to the reduction of tissue damage caused by inflammation. Furthermore, cashew nut supplementation resulted in a significant increase in the antioxidant markers SOD, CAT, and GSH, which had been reduced by carrageenan injection. These increases were statistically significant (*p* < 0.01), indicating an improvement in antioxidant defense after cashew nut administration.

Finally, regarding inflammatory cytokine synthesis, the administration of cashew nuts led to a significant reduction in pro-inflammatory cytokines, such as TNF-α, IL-1β, and IL-6, compared to the CAR group. There was also a notable increase in the production of the anti-inflammatory cytokine IL-10 (*p* < 0.001 vs. CAR). Histological analysis revealed that cashew nuts significantly reduced cellular infiltration and edema formation in paw tissues compared to the CAR group, which exhibited severe disruption of tissue architecture. This effect further confirmed the protective role of cashew nuts in mitigating inflammation. In conclusion, cashew nuts, at a dose of 100 mg/kg, significantly reduced inflammation, oxidative stress, and pain in the experimental model, demonstrating their potential as a natural anti-inflammatory and antioxidant agent. According to Table 2.

**Figure 2:**
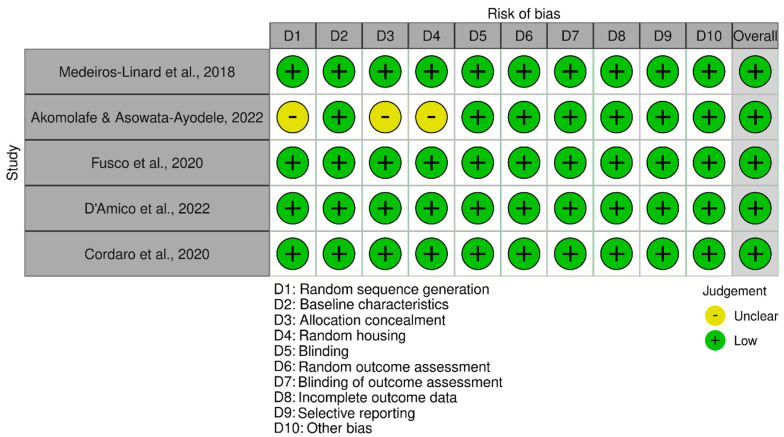
Systematic Review Center for Laboratory Animal Experimentation (RoB/SYRCLE). The corresponding citations presented in Figure 2: Medeiros-Linard et al. (2018) [10]; Akomolafe, Abiola, and Asowata-Ayodele (2022) [11]; Fusco et al. (2020) [12]; D’Amico et al. (2022) [13]; Cordaro et al. (2020) [14].

Regarding the bias risk for the corresponding studies in the above-mentioned table, we observed that the RoB/Syrcle tool was used, which contains 10 domains. All the studies included in the table resulted in a low risk of bias assessment. Please see Figure 2 for more details.

## 4. Discussion

This comprehensive systematic review was conducted with the aim of providing an overview of research exploring cashew nut consumption and its potential effects on oxidative stress. Although the clinical data included in this study are limited, the authors reviewed experimental studies to present a broad perspective on the impacts of cashew nuts, both in their whole form and as isolated compounds, with the intention of facilitating understanding and better synthesizing the evidence regarding their effects on oxidative stress.

To deepen knowledge about the bioactive markers present in cashew nuts, a comprehensive analysis was performed to identify and categorize these substances. This investigation aims to clarify the actions and effects of the bioactive components in cashew nuts, contributing to a clearer and more grounded understanding of their properties and potential health benefits. As part of a summary on cashew nuts and their compounds, we refer to the dietary analysis conducted by Salehi et al. [1], which sought to explore the chemical, nutritional, and biotechnological composition of plant species in the Anacardium genus. Bioactive compounds present in cashew nuts and similar species were identified. The study analyzed eleven samples of cashew nuts from various countries, including India, Brazil, Côte d’Ivoire, and countries in the Indochinese Peninsula.

The evaluated nutritional composition included total dietary fiber, sugar, protein, lipid profile, sodium, and energy content. Cashew nuts demonstrated a total fat concentration equivalent to 48.3% of their total weight, with 79.7% unsaturated fatty acids, 20.1% saturated fatty acids, and 0.2% trans fatty acids. Regarding protein content, cashew nuts contain a concentration of 21.3 g/100 g, making it the second-largest component. The carbohydrate concentration was 20.5 g/100 g. The average sodium and energy content in cashew nuts were 144 mg/kg and 2525 kJ/100 g, respectively.

It is important to emphasize that these concentrations may vary depending on the soil conditions where *Anacardium occidentale* L. was cultivated. Furthermore, variations in concentration can directly influence the potential of the nut. Based on results from rodent studies, it is evident that compounds present in cashew nuts act as important controllers of lipid peroxidation while also significantly enhancing antioxidant defenses. In this context, the review by Alasalvar and Bolling [2] indicates that cashew nuts possess a rich phytochemical composition, with significant concentrations of total phenols, polyphenols, alkylphenols, and phenolic acids. Additionally, they are abundant in flavonoids, stilbenes, and phytates. Cashew nuts also contain procyanidins, gallic acid, gallotannins, ellagic acid, and ellagitannins. Regarding lipophilic substances, the presence of tocopherols, phytosterols, sphingolipids, and carotenoids is noteworthy. Furthermore, the presence of vitamins B, C, and E, as well as minerals such as Na, K, Ca, Mg, P, Fe, Cu, and Se, has been identified in *Anacardium* species. Concentrations of ascorbic acid (34.2 mg/100 g), thiamine (15.5 mg/100 g), riboflavin (2.90 mg/100 g), and niacin (0.23 mg/100 g) have also been reported [1].

Following this explanation of the bioactive compounds present in cashew nuts and considering their potential for oxidative stress control and the strengthening of antioxidant defenses, it is important to highlight not only the benefits of these compounds but also the appropriate consumption quantity to achieve such effects. On the other hand, it is crucial to consider that, as a food with high caloric value and rich in lipids, moderate consumption is recommended, with portions of up to 100 g daily, to ensure positive outcomes without adverse health effect [15,16].

Moreover, after this brief review of the active compounds in cashew nuts and their potential effects on oxidative stress control and the enhancement of antioxidant defenses, it is relevant to highlight the role of the plant *Anacardium occidentale* L. as a source of these compounds. Based on this, the relationship between cashew nut consumption, oxidative stress, antioxidant defenses, and the inflammatory process will be discussed, exploring how these elements interact and contribute to health promotion.

It is important to note that the results presented in the systematic review demonstrate significant benefits associated with cashew nut consumption. Variations in dosages related to oxidative stress effects, ranging from 25 to 100 mg/kg of cashew nuts, should also be considered. Most studies included in the review utilized raw or roasted cashew nuts, while others employed isolated compounds, justifying the use of lower doses due to the concentrated nature of these compounds.

An important similarity among the included experiments is the dose of 100 mg/kg of cashew nuts, which was shown to be effective in most studies. Although different types of pathological alterations, treatment periods, and pathology-inducing compounds were addressed, all experiments converged on the reduction of oxidative stress with cashew nut supplementation at this dosage. It is worth noting that isolated compounds from cashew nuts, such as anacardic acids (AAs), also showed benefits in controlling oxidative stress, with dosage variations ranging from 1 to 100 mg/kg.

Supported by these findings, the studies by Medeiros-Linard et al. [10] investigated oxidative stress reduction in experimental models of Parkinson’s disease supplemented with anacardic acids (AAs), isolated compounds from cashew nuts. AAs were administered in doses ranging from 1 to 100 mg/kg/day, with reductions in lipid peroxidation observed at doses of 25 to 100 mg/kg/day. Analyses conducted in the basal ganglia of the nervous system indicated a significant impact on reducing oxidative stress.

Similarly, the in vitro study conducted by Augusto et al. [15] evaluated the efficacy of 50 mg/kg of purified anacardic acids (AAs), extracted from cashew nut shell liquid, at different stages of oxidative stress and inflammation induced by rotenone in the substantia nigra (SN). The study included mice divided into four groups: control, AA (isolated compound from cashew nuts), AA + rotenone, and rotenone. Oxidative stress levels were assessed through nitric oxide and glutathione fractions, as well as molecules related to DNA/RNA repair and inflammatory cytokines. The groups receiving cashew nut compounds showed a reversal of rotenone’s effects on oxidative stress, with a significant increase in DNA repair molecules. The study concluded that AAs represent a promising approach for combating oxidative and inflammatory conditions induced by rotenone.

Although methodological differences and heterogeneity in approaches were noted, the studies indicated that the AAs present in cashew nuts and the *Anacardium occidentale* L. plant have a significant effect on reducing oxidative stress. In the studies conducted by Medeiros-Linard et al. [10], AAs were administered in pure and isolated forms, with effective doses ranging from 25 to 100 mg/kg/day. These promising results suggest that cashew nuts and their isolated compounds, such as anacardic acids, may be valuable allies in managing oxidative stress, particularly in neurodegenerative contexts such as Parkinson’s disease.

Corroborating the aforementioned data, but employing heterogeneous methods compared to those previously described, are the experiments conducted by Gomes Júnior et al. [16]. These studies aimed to evaluate the impact of anacardic acid (AA), isolated from cashew nut shell liquid, on anxiety treatment and its effects on oxidative stress in mice. Behavioral tests were used to assess anxiety-related effects, and flumazenil was employed to determine the level of involvement of the GABA system in AA’s action. Regarding oxidative stress, malondialdehyde levels were evaluated alongside substances from the glutathione family, followed by measurements of catalase activity. Notably, with oral doses ranging from 25 to 50 mg/kg, the animals demonstrated improvement in anxiety symptoms, as well as a reduction in oxidative stress, accompanied by increased antioxidant defenses, as indicated by the aforementioned markers.

Therefore, the results suggest that isolated compounds from cashew nuts, as well as the *Anacardium occidentale* L. plant, have the potential to reduce oxidative stress and enhance antioxidant defenses, as observed in the discussed studies. These findings indicate that these compounds may have important therapeutic applications, especially in conditions associated with oxidative stress and inflammation.

In the context of consuming raw or roasted cashew nuts administered orally without isolated compounds, a dosage of 100 mg/kg again demonstrated significant improvements in oxidative stress, antioxidant capacity, and inflammation, as evidenced by the systematic review results. Supporting these findings, experiments conducted by Akomolafe, Oyeleye, and Oboh [17] evaluated the effects of 10% and 20% roasted cashew nuts on reproductive hormones, sperm parameters, testicular and epididymal antioxidant status, and steroidogenic enzyme activities in cisplatin (CP)-induced rats. A reduction in reactive species levels was observed in the testes and epididymis of CP-induced rats fed roasted cashew nuts compared to untreated CP-induced rats.

Although data on the benefits of roasted cashew nuts remain limited, the reduction in oxidative stress is promising. However, there is still no clear scientific consensus on these effects. This field holds great potential for future exploration, particularly concerning redox balance.

In line with this, experiments conducted by Fusco et al. [12] demonstrated that the administration of cashew nuts (100 mg/kg) significantly reduced mortality rates, blood pressure drops, and oxidative stress, in addition to restoring the activities of antioxidant enzymes through NRF2 and NF-κB pathways. Treatment with cashew nuts reduced plasma cytokine levels, nitrotyrosine and PARP expression, as well as adhesion molecule expression.

It is important to highlight that the NRF2 pathway is a physiological mechanism for controlling cellular oxidative stress. Under normal conditions, NRF2 is located in the cytoplasm, bound to the KEAP1 protein, which promotes its degradation. However, when there is an increase in the production of reactive oxygen species (ROS) or other stressors, NRF2 dissociates from KEAP1 and translocates to the cell nucleus. There, it binds to antioxidant response elements (AREs), activating the transcription of genes encoding antioxidant enzymes, such as superoxide dismutase (SOD), catalase, and glutathione peroxidase (GPx) [18,19,20,21,22,23,24].

Furthermore, it is worth noting that cashew nuts, through their bioactive compounds, enhance this pathway. These compounds boost the physiological response to oxidative stress by regulating the expression of genes encoding antioxidant defenses and detoxifying substances. As a result, this physiological cascade promotes the neutralization of reactive oxygen species (ROS), protecting cells against oxidative damage [12,22].

Additionally, another pathway contributing to these benefits involves NF-κB. NF-κB generally remains inactive in the cellular cytoplasm, bound to a substance known as IκB (Inhibitor of kappa B). In situations of stimulation by pro-inflammatory agents or reactive oxygen species, NF-κB is activated, triggering signaling that leads to the phosphorylation and degradation of IκB. Through the previously described pathway, NF-κB can induce the expression of antioxidant enzymes, such as superoxide dismutase (SOD), catalase (CAT), and glutathione peroxidase (GPx) [25].

On the other hand, there is a less commonly studied pathway for activating endogenous antioxidant enzymes, which involves bioactive compounds found in cashew nuts, such as vitamin E (associated with protection against lipid peroxidation), selenium (which enhances glutathione peroxidase activity), and zinc, which is directly related to stimulating enzymatic antioxidant capacity. However, this pathway remains underexplored regarding its physiological mechanisms [26,27].

Studies suggest that NF-κB and NRF2 do not act in isolation but rather in a complex and interdependent relationship between cellular signaling mechanisms and their respective biological pathways. While NF-κB is widely recognized for its role in controlling inflammatory processes, its activation contributes to the gene expression of antioxidant enzymes. Regarding emerging mechanisms, studies included in this review emphasize that bioactive compounds from cashew nuts modulate both NRF2 and NF-κB through different pathways, highlighting NRF2 activation as the primary mechanism for increasing antioxidant capacity. However, the relationship between these pathways remains an open field for investigation. It is possible that the simultaneous activation of NRF2 and NF-κB allows for a coordinated response to oxidative and inflammatory stress, optimizing the protective effects of cashew nuts [25,27,28].

Corroborating these findings, experiments conducted by Siracusa et al. [18] evaluated the anti-inflammatory and antioxidant potential of orally administered cashew nuts in rodents with colitis. Similar to other experiments, a dosage of 100 mg/kg was used in animal models. Colitis was induced in the animals using dinitrobenzene sulfonic acid. It was observed that animals with colitis receiving cashew nut supplementation showed reduced oxidative stress and infiltration of anti-inflammatory cytokines.

Finally, experiments conducted by Cordaro et al. [14] demonstrated that cashew nuts significantly reduced paw volume and carrageenan-induced pain, as well as decreased MPO and MDA activity in paw tissues, nitrite/nitrate levels in paw exudate, and inflammatory cytokines (interleukins and tumor necrosis factor). There was also an increase in interleukin-10 levels. It is important to emphasize the significant enhancement in antioxidant defenses.

Thus, we observe that nuts, in general, have a potential effect in reducing oxidative stress, inflammatory processes, and enhancing antioxidant defenses, owing to their phenolic content. Furthermore, they are a widely accepted supplement among individuals and provide beneficial effects both in the short and long term.

It is worth noting that one of the most evident factors in the studies included in this research is the heterogeneity of the studies, as well as the inclusion of experiments conducted on both animals and humans. This demonstrates the potential of cashew nuts to reduce oxidative stress through oral ingestion. In animal studies, benefits were observed with doses of 100 mg/kg/day, whereas in humans, benefits were evident with a mixture of approximately 30 g of Brazil nuts. Additionally, cashew nuts showed secondary beneficial effects, particularly in controlling inflammatory processes, as observed in the analyzed experiments.

This systematic review also highlights some limitations, primarily related to the variability of the experiments found in the databases. This variability limits the observation of a consistent potential effect on oxidative stress, antioxidant defenses, and inflammatory process control.

Although the results are promising, the heterogeneity of the analyzed studies prevents definitive conclusions from being drawn. It is essential that future research be conducted with more homogeneous and standardized methodologies to enable a clearer understanding. In light of this, we can conclude that nuts, in general, have a potential effect in reducing oxidative stress, controlling inflammatory processes, and enhancing antioxidant defenses due to their phenolic content.

## 5. Conclusions

Cashew nuts have significant potential in reducing oxidative stress and controlling the inflammatory process. Evidence suggests that the bioactive compounds present in cashew nuts, such as anacardic acids, play an important role in improving redox balance and protecting against oxidative damage in various experimental models. However, the significant variability in the methods and materials used, dosages, routes of administration, isolated substances and other compounds from cashew nuts, tissues studied, experimental period, and study groups prevents the formulation of definitive conclusions about the efficacy of cashew nuts in controlling oxidative stress.

Future studies should focus on more homogeneous and standardized methodologies to robustly validate the benefits of cashew nuts. In particular, well-designed clinical trials in humans are essential to confirm the effects observed in animal models and to investigate the clinical applicability of the bioactive compounds in cashew nuts. Additionally, exploring the molecular mechanisms underlying the antioxidant and anti-inflammatory actions of cashew nuts can provide valuable insights for developing effective therapeutic interventions.

The main limitations of this systematic review include the scarcity of literature on the relationship between cashew nuts and oxidative stress, as well as the significant heterogeneity of the studies analyzed. This variability contributes to observing only a potential effect of cashew nuts on oxidative stress, antioxidant defenses, and the control of the inflammatory process. Moreover, the inclusion of studies with different experimental models, dosages, and routes of administration makes direct comparison of the results difficult. Despite these limitations, the results are promising and highlight the need for more research to better understand the effects of cashew nuts and their isolated compounds on oxidative stress and inflammation. This will enable a more consistent validation of the benefits of this intervention and its potential clinical applications.

## Figures and Tables

**Table 1:** Search strategy.

#1 “Anacardium” OR (Anacardiums) OR (*Anacardium occidentale*) OR (*Anacardium occdentales*) OR (occidentales, Anacardium) OR (Cashew) OR (Cashews) #2 “Oxidative Stress” OR (Oxidative Stresses) OR (Stress, Oxidative) OR (Antioxidative Stress) OR (Antioxidative Stresses) OR (Stress, Antioxidative) OR (Anti-oxidative Stress) OR (Anti oxidative Stress) OR (Anti-oxidative Stresses) OR (Stress, Anti-oxidative) OR (Oxidative Damage) OR (Damage, Oxidative) OR (Oxidative Damages) OR (Oxidative Stress Injury) OR (Injury, Oxidative Stress) OR (Oxidative Stress Injuries) OR (Stress Injury, Oxidative) OR (Oxidative Injury) OR (Injury, Oxidative) OR (Oxidative Injuries) OR (Oxidative Cleavage) OR (Cleavage, Oxidative) OR (Oxidative Cleavages) OR (Oxidative DNA Damage) OR (DNA Damage, Oxidative) OR (Damage, Oxidative DNA) OR (Oxidative DNA Damages) OR (DNA Oxidative Damage) OR (DNA Oxidative Damages) OR (Damage, DNA Oxidative) OR (Oxidative Damage, DNA) OR (Oxidative and Nitrosative Stress) OR (Oxidative Nitrative Stress) OR (Nitrative Stress, Oxidative) OR (Oxidative Nitrative Stresses) OR (Stress, Oxidative Nitrative) OR (Nitro-Oxidative Stress) OR (Nitro Oxidative Stress) OR (Nitro-Oxidative Stresses) OR (Stress, Nitro-Oxidative) OR (Stresses, Nitro-Oxidative) #3 “Rats” OR (Rat) OR (Rattus) OR (Rattus norvegicus) OR (Rats, Norway) OR (Rats, Laboratory) OR (Laboratory Rat) OR (Laboratory Rats) OR (Rat, Laboratory)

**Table 2:** Compendium of selected articles.

Authors (Year) [Reference]	Objective of the Study	Experimental Groups (n of Animals/Group)	Substances Administered (Doses, Route, Period, Administration Order, and Interval)	Analyses Conducted	Significant Results (Values and Statistical Significance)
Medeiros-Linard et al. (2018) [10]	To evaluate the neuroprotective effects of anacardic acids against rotenone-induced neurotoxicity in a Parkinson’s disease rat model.	Adult male Wistar rats (6–10 animals per group): Control, Rotenone, and Rotenone + increasing doses of anacardic acids.	Rotenone: 3 mg/kg/day, subcutaneous (s.c.), for 5 consecutive days. Anacardic acids: 1–100 mg/kg/day, oral, administered 1 h prior to rotenone. Interval: 1 h.	Behavioral tests (open field, rotarod, elevated T-maze), lipoperoxidation, total SOD activity, and gene expression (mitochondrial and cytoplasmic) in nigra, striatum, and cortex.	Reduction in lipoperoxidation (*p* < 0.05) and an increase in total SOD activity at doses ≥25 mg/kg (*p* < 0.01). Improvements in motor and cognitive performance at doses ≥10 mg/kg (*p* < 0.05).
Akomolafe, Abiola, and Asowata-Ayodele (2022) [11]	To investigate the effects of a roasted cashew nut-enriched diet against cisplatin-induced brain damage.	Six groups: Control, Cisplatin, Cisplatin + 10% cashew, Cisplatin + 20% cashew, 10% cashew, and 20% cashew.	Cisplatin: 7 mg/kg, intraperitoneal (i.p.), single dose. Cashew nut: Diet containing 10% or 20% cashew, administered for 28 days. Order: Cashew-enriched diet provided before and during cisplatin treatment.	Activity of SOD, GPx, GST; levels of ROS and TBARS; brain histology; cholinesterase activity (AChE, BChE).	Increase in SOD, GPx, and GST activity (*p* < 0.01). Reduction in ROS and TBARS levels (*p* < 0.05). Prevention of neuronal damage (*p* < 0.01).
Fusco et al. (2020) [12]	To evaluate the antioxidant effects of cashew nuts in intestinal ischemia/reperfusion injuries in rats.	Four groups: Sham, Vehicle, I/R, and I/R + Cashew (10 rats/group).	Cashew nut: 100 mg/kg/day, oral, administered before and during ischemia/reperfusion (30 min of ischemia, 1 h of reperfusion).	Inflammatory markers (ICAM-1, P-selectin), oxidative stress markers (ROS, nitrotyrosine, MPO), intestinal histology, arterial pressure, and mortality.	Reduction in mortality (*p* < 0.001). Increase in antioxidant activity (*p* < 0.01). Reduction in inflammation and tissue damage (*p* < 0.05).
D’Amico et al. (2022) [13]	To analyze the antioxidant and anti-inflammatory effects of cashew nuts in a hyperhomocysteinemia model.	Four groups: Sham + Vehicle, Sham + Cashew, Methionine + Vehicle, Methionine + Cashew (10 rats/group).	Methionine: 1 g/kg/day, oral, for 30 days. Cashew nut: 100 mg/kg/day, oral, administered concomitantly with methionine. Order: Cashew administered daily alongside methionine.	Inflammatory markers (TNF-α, IL-6), MDA, GSH levels; histology of the liver, kidneys, and colon.	Reduction in MDA and inflammatory cytokines (*p* < 0.01). Improvement in GSH levels (*p* < 0.05). Decrease in histological damage (*p* < 0.05).
Cordaro et al. (2020) [14]	To evaluate the anti-inflammatory and antioxidant effects of cashew nuts in carrageenan-induced paw edema in rats.	Three groups: Control, Carrageenan, and Carrageenan + Cashew Nut (10 rats/group).	Carrageenan: 0.1 mL of a 1% solution, subcutaneous (s.c.), in the right hind paw. Cashew nut: 100 mg/kg, oral, administered 30 min prior to carrageenan. Order: Cashew administered before carrageenan.	Paw volume (plethysmometry), MPO, MDA, ROS, inflammatory cytokines (TNF-α, IL-1β), pain-related tests (Von Frey and plantar test).	Reduction in paw edema (*p* < 0.01) and MPO levels (*p* < 0.05). Significant increase in antioxidant activity (*p* < 0.01). Improvement in inflammatory response and reduction in pain (*p* < 0.05).
